# Therapeutic Potential of Glucagon-like Peptide-1 Receptor Agonists in Obstructive Sleep Apnea Syndrome Management: A Narrative Review

**DOI:** 10.3390/diseases12090224

**Published:** 2024-09-23

**Authors:** Silvano Dragonieri, Andrea Portacci, Vitaliano Nicola Quaranta, Pierluigi Carratu, Zsofia Lazar, Giovanna Elisiana Carpagnano, Andras Bikov

**Affiliations:** 1 Respiratory Medicine, Dipartimento di Biomedicina Traslazionale e Neuroscienze, University of Bari, 70121 Bari, Italy; a.portacci01@gmail.com (A.P.); vitalianonicola.40@gmail.com (V.N.Q.); elisiana.carpagnano@uniba.it (G.E.C.); 2 Internal Medicine “A. Murri”, Department DIMEPREJ, University of Bari, 70121 Bari, Italy; pierluigi.carratu@uniba.it; 3 Department of Pulmonology, Semmelweis University, 1085 Budapest, Hungary; lazar.zsofia@med.semmelweis-univ.hu; 4 Wythenshawe Hospital, Manchester University NHS Foundation Trust, Manchester M13 9WL, UK; andras.bikov@gmail.com

**Keywords:** Obstructive Sleep Apnea syndrome, OSAS, GLP-1 receptor agonists, weight loss, respiratory control

## Abstract

**Background:** Obstructive Sleep Apnea (OSA) is a prevalent disorder characterized by repetitive upper airway obstructions during sleep, leading to intermittent hypoxia and sleep fragmentation. Current treatments, particularly Continuous Positive Airway Pressure (CPAP), face adherence challenges, necessitating novel therapeutic approaches. **Methods**: This review explores the potential of Glucagon-like Peptide-1 receptor agonists (GLP-1RA), commonly used for type 2 diabetes and obesity, in managing OSA. GLP-1RA promotes weight loss, enhances insulin sensitivity, and exhibits anti-inflammatory and neuroprotective properties, potentially addressing key pathophysiological aspects of OSA. **Results**: Emerging evidence suggests that these agents may reduce OSA severity by decreasing upper airway fat deposition and improving respiratory control. Clinical trials have demonstrated significant reductions in the Apnea-Hypopnea Index (AHI) and improvements in sleep quality with GLP-1 therapy. **Conclusions:** Future research should focus on elucidating the mechanisms underlying GLP-1 effects on OSAS, optimizing combination therapies, and identifying patient subgroups that may benefit the most. Integrating GLP-1RA into OSAS management could revolutionize treatment by addressing both the metabolic and respiratory components of the disorder, ultimately enhancing patient outcomes.

## 1. Obstructive Sleep Apnea

Obstructive Sleep Apnea (OSA) is a common disorder characterized by repetitive episodes of complete or partial collapse of the upper airway during sleep. These events may cause intermittent hypoxia and fragmented sleep [1]. It is estimated that OSA affects one billion people. Around 2–9% of adults suffer from its symptomatic form, called Obstructive Sleep Apnea syndrome (OSAS). The prevalence increases in men, older adults, and individuals with obesity [2]. Further risk factors include anatomical abnormalities, family history, and certain medical conditions such as heart failure, hypothyroidism, and acromegaly [1].

The pathophysiology of OSA involves both anatomical and functional factors, leading to upper airway collapse during sleep [3]. The anatomical factors include a narrow or collapsible airway, enlarged tonsils, or a large tongue. Craniofacial abnormalities such as retrognathia, maxillary hypoplasia, or a narrow maxilla can reduce airway patency, thereby increasing the risk of airway obstruction during sleep [4]. Mandibular advancement devices (MADs) are often employed in such cases as they work by advancing the mandible and expanding the airway [5]. Functional factors include reduced neuromuscular control of the airway muscles, low arousal threshold, and high loop gain [6], leading to respiratory instability. The repetitive obstruction results in cycles of hypoxia and reoxygenation, which contribute to oxidative stress and systemic inflammation. The resulting sleep fragmentation and intermittent hypoxia have profound effects on various organ systems, increasing the risk of cardiovascular diseases, metabolic disorders, and neurocognitive impairments [3]. Recent studies have highlighted the role of inflammation, oxidative stress, and hypoxia as key mediators that link OSAS with various comorbidities, such as cardiovascular disease, diabetes, and neurocognitive disorders [7,8]. Emerging evidence has also underscored the relationship between OSAS and metabolic syndrome, with shared mechanisms such as systemic inflammation and insulin resistance. Moreover, chronic intermittent hypoxia, a hallmark of OSAS, contributes to endothelial dysfunction and the progression of atherosclerosis, thus significantly increasing the risk of hypertension, stroke, and myocardial infarction [9]. Understanding these underlying mechanisms is essential for developing comprehensive treatment strategies that address both OSAS and its associated comorbidities.

Continuous Positive Airway Pressure (CPAP) therapy is the gold standard treatment for OSA [10]. It works by providing a constant stream of air through a mask, keeping the airway open during sleep. However, despite its effectiveness, patient adherence to CPAP therapy is often a challenge due to discomfort, noise, and the inconvenience of wearing a mask [10,11]. Further treatments include mandibular advancement devices, positional therapy, hypoglossal nerve stimulation, and surgical procedures such as adenotonsillectomy or orofacial surgery. Whilst they are effective in reducing disease severity and improving daytime symptoms, it is yet to be proven as to whether they modify the risk of comorbidities [12].

Lifestyle changes are therefore crucial components of OSA management. Weight loss, avoidance of alcohol, sedatives, and smoking are recommended, as these factors can exacerbate OSAS [13]. Most particularly, weight loss is associated with a significant improvement in the severity of OSA [14]. Moreover, compared to or combined with CPAP, weight loss leads to better cardiovascular and metabolic outcomes in patients with OSA [15]. Although multiple medications were trialed, there is no pharmacological treatment currently available to treat OSA [16]. However, recent trials with glucagon-like peptide-1 receptor agonists (GLP-1RAs) in OSA show promising results providing potential for pharmacological management of OSA [17].

The aim of this narrative review is to explore the therapeutic potential of GLP-1RAs in the management of OSA, by summarizing available preclinical and clinical evidence on their efficacy, mechanisms, and potential impact on OSA comorbidities.

## 2. Glucagon-like Peptide-1

Glucagon-like Peptide-1 (GLP-1) is a 30-amino acid peptide hormone derived from the transcription product of the proglucagon gene [18]. It is primarily secreted by the L-cells of the small intestine in response to nutrient ingestion. GLP-1 exists in two main forms: GLP-1(7–37) and GLP-1(7–36) amide, with the latter being the predominant active form in humans [18]. GLP-1 is an incretin hormone produced in the intestines in response to nutrient intake. Whilst the main trigger is glucose [19], lipids can also induce GLP-1 production [20]. It is important to notice that GLP-1 production is reduced in obese individuals [21]. However, the studies on the effect of hypoxia are contradictory [22,23]. In addition, inflammation may block GLP-1 release [24]. Nevertheless, Matsumoto et al. reported elevated GLP-1 levels in OSA [25]. However, the contradictory effects of obesity, hypoxia, and inflammation may suggest a high variation in plasma GLP-1 concentrations in OSA depending on the phenotype.

GLP-1 exerts its effects through binding to the GLP-1 receptor (GLP-1R), a G-protein-coupled receptor expressed in multiple tissues, including the pancreas, brain, heart, and gastrointestinal tract [18,21]. Upon binding to GLP-1R, the following intracellular pathways are activated: (1) the activation of adenylyl cyclase increases cAMP levels, which activate protein kinase A (PKA). This pathway enhances insulin secretion and inhibits glucagon release. (2) GLP-1 signaling also activates phosphatidylinositol-3-kinase (PI3K) and protein kinase B (Akt), which are involved in promoting cell survival and insulin gene expression. (3) The mitogen-activated protein kinase (MAPK) pathway is stimulated by GLP-1R activation, contributing to beta-cell proliferation and differentiation [18,26]. GLP-1 enhances insulin secretion from pancreatic beta cells, inhibits glucagon release by alpha cells, slows gastric emptying via inhibiting the vagal nerve, and promotes satiety. These actions make GLP-1 a critical regulator of glucose homeostasis and appetite.

GLP-1 receptor agonists (GLP-1RA) are a class of medications that mimic the effects of endogenous GLP-1. They are primarily used to treat type 2 diabetes and obesity. Commonly used GLP-1 receptor agonists include exenatide, liraglutide, dulaglutide, semaglutide and tirzepatide. Tirzepatide simultaneously targets both incretin receptors, including the glucose-dependent insulinotropic polypeptide receptor (GIPR) and GLP-1R, contributing to a greater glycemic and weight effect [21]. These medications improve glycemic control, promote weight loss, and have additional cardiovascular and neuroprotective benefits [21,27,28,29]. Potential risks associated with their prolonged use may include gastrointestinal disturbances, including nausea, vomiting, diarrhea, and constipation [21,27,28,29].

**Figure 1:**
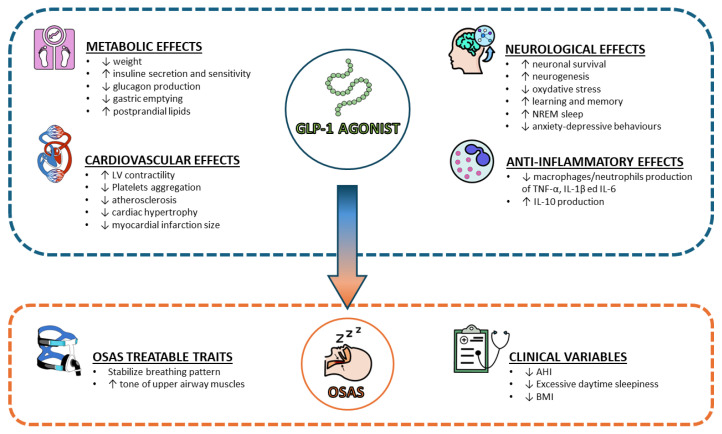
Main effects of GLP-1 agonist on several aspects of OSAS pathophysiology. Abbreviations: GLP-1 = Glucagon-like Peptide-1; OSAS = Obstructive Sleep Apnea Syndrome; AHI = Apnea-Hypopnea Index; BMI = Body Mass Index; TNF = Tumor Necrosis Factor; IL = Interleukin; LV = Left Ventricle; NREM = Non-rapid eye movement.

Taking into account the beneficial role of GLP-1 and GLP-1RA in obesity and diabetes; research has been focused on their roles in OSA. This review will focus on the pre-clinical findings on GLP-1 in OSA and will review the observational and randomized controlled studies published with GLP-1RA in OSA (Figure 1).

## 3. The Pathophysiology of OSA, Focusing on the Role of GLP-1

### 3.1. Upper Airway Collapse and Respiratory Control

During sleep, especially during rapid eye movements (REM) sleep, there is a natural reduction in the airway dilator muscle activity, such as the genioglossus muscle [3]. As a result, the caliber of the upper airways is narrower in sleep than in wakefulness. In addition, due to pressure changes in the pharyngeal airways, during inspiration, the airways tend to collapse [30]. However, this physiological phenomenon is more pronounced in patients with OSA, partly due to the increased upper airway tissue weight, but more importantly, due to inadequate dilator muscle activity [31]. Moreover, instability in the control of breathing, such as an exaggerated ventilatory response to hypoxia and hypercapnia, can lead to cyclical patterns of airway obstruction. This instability is characterized by alternating periods of apnea and hyperventilation, which can perpetuate the cycle of airway collapse and arousal [1].

GLP-1 receptors are expressed in the central nervous system, including areas involved in respiratory control. The activation of these receptors can influence respiratory patterns and stability. Preclinical studies suggest that GLP-1 receptor activation may enhance respiratory drive and stabilize breathing patterns [18,29]. GLP-1 may also affect the tone of upper airway muscles. By influencing neuromuscular control, GLP-1 receptor agonists could help prevent the collapse of the upper airway during sleep, thereby reducing the frequency and severity of apneas and hypopneas [29].

### 3.2. Appetite and Weight Loss

Obesity is a major risk factor for OSA [32]. The association between OSA and obesity is bidirectional. On one hand, OSA leads to physical inactivity, increased calorie intake, and metabolic dysfunction, contributing to the development of obesity [33]. On the other hand, increased fat deposition around the neck and upper airway can compress the airway and reduce its patency [1,3], and visceral fat contributes to reduced lung volumes, which can decrease the caudal traction on the upper airway, increasing its collapsibility [1,3,34]. Patients with OSA tend to consume high-calorie, glucose-, and lipid-rich diets [35]. Both sleep fragmentation and chronic intermittent hypoxia contribute to appetite dysregulation in OSA [36].

GLP-1 promotes weight loss by slowing gastric emptying and regulating appetite. These combined effects lead to significant weight loss in patients treated with GLP-1 receptor agonists [18]. By slowing gastric emptying [37], GLP-1 causes the feeling of fullness and reduces overall food intake. GLP-1 receptors are distributed in the central nervous system, most particularly in the appetite-regulating parts of the hypothalamus [38]. These central receptors are complemented by the peripheral GLP-1 receptors located in the autonomic nervous system and nodose ganglion [39]. Studies show that the appetite-suppressing effect of GLP-1RAs is mediated through these receptors [38,40]. In addition, intestinal GLP-1 acts on sensory nerves and via the parabrachial nucleus contributes to meal termination [41].

Numerous clinical trials have demonstrated the efficacy of GLP-1RA in inducing weight loss. For example, studies on liraglutide and semaglutide have shown substantial reductions in body weight in both diabetic and non-diabetic populations [42,43]. These weight losses are clinically meaningful and have been associated with improvements in various obesity-related conditions, including OSA. Weight loss is a critical component in managing OSA, particularly in obese patients. By reducing adipose tissue around the upper airway, weight loss decreases the mechanical load on the airway, reducing its tendency to collapse during sleep. From this perspective, GLP-1RA-induced weight loss could lead to improvements in the severity of OSA [44].

### 3.3. Glucose Metabolism and Insulin Sensitivity

OSA is strongly associated with metabolic disorders such as insulin resistance and type 2 diabetes [1,3]. Several mechanisms lead to insulin resistance in OSA, including hypoxia, sleep fragmentation, sympathetic nervous system activation, dyslipidemia, oxidative stress, and inflammation [45,46,47]. In turn, insulin resistance contributes to dyslipidemia [48], hyperglycemia [49], and cardiovascular consequences [50,51] in OSA.

GLP-1 plays a crucial role in glucose metabolism and homeostasis [26]. GLP-1 enhances glucose-dependent insulin secretion from pancreatic beta cells, thereby improving postprandial glucose control. It also inhibits glucagon release from alpha cells, reducing hepatic glucose production, and slows down gastric emptying, promoting satiety [26]. These actions help maintain blood glucose levels within the normal range, particularly in individuals with type 2 diabetes. GLP-1RAs improve insulin sensitivity by reducing systemic inflammation and oxidative stress, both of which are implicated in insulin resistance [26]. Additionally, weight loss induced by GLP-1RA further enhances insulin sensitivity. Finally, GLP-1Ras reduce postprandial lipid levels via attenuating chylomicron production [52], contributing to improving insulin resistance.

### 3.4. Anti-Inflammatory Effects

Chronic intermittent hypoxia and sleep fragmentation in OSA lead to systemic and local inflammation [1]. On one hand, inflammation can cause edema of the upper airway tissues, further narrowing the airway and exacerbating the tendency for collapse [34]. On the other hand, inflammation in end organs contributes to their dysfunction, insulin resistance, atherosclerosis, and sympathetic system activation [53].

GLP-1RAs exhibit significant anti-inflammatory effects [54,55]. GLP-1 receptors are present in monocytes, neutrophils, and lymphocytes [55]; therefore, it is not surprising that GLP-1RAs reduce the production of pro-inflammatory cytokines such as TNF-α, IL-1β, and IL-6 in macrophages and neutrophils [56,57]. In addition, GLP-1 increases the production of anti-inflammatory IL-10 [58]. Oxidative stress is a major hallmark of OSA. Semaglutide was shown to prevent oxidative stress-induced myocardial injury in rats [59]. This modulation of the immune response could help reduce systemic inflammation in OSA, potentially preventing end-organ damage.

### 3.5. Cardiovascular Risk

OSA significantly increases the risk of cardiovascular diseases, including hypertension, coronary artery disease, heart failure, and stroke [60]. The linking mechanisms include hypoxia, oxidative stress, vascular inflammation, dyslipidemia, increased sympathetic activity, hypercoagulation, and endothelial dysfunction [1,48,54,61]. As a result, moderate to severe OSA is a risk factor for cardiovascular mortality in the general population [62] and patients with increased cardiovascular risk [63].

GLP-1Rs are widely distributed in cardiac myocytes and endothelial cells [64]. In addition, GLP1 receptors in the area postrema are responsible for autonomic regulation of blood pressure and heart rate [65].

The beneficial role of GLP-1RA has been summarized recently [66]. In animal models, treatment with GLP-1RA has resulted in improvement in vascular inflammation and atherosclerosis [67]. In the experimental hypertension model, GLP-1RA resulted in the amelioration of cardiac hypertrophy [68]. In patients post myocardial infarct, GLP-1 infusion resulted in improvement in systolic function [69], and treatment with GLP1RAs resulted in decreased myocardial infarct size [70,71] and improvement in left ventricular function [72]. Liraglutide was also shown to attenuate platelet aggregation [73]. In patients with diabetes, GLP-1Ras were shown to prevent major cardiovascular events [74].

### 3.6. Neurological Effects

Chronic sleep disruption in OSA affects various neurocognitive functions. Patients with OSA often experience impairments in attention, memory, executive function, and mood [3]. The underlying mechanisms include the direct effects of intermittent hypoxia on neuronal health, as well as the indirect effects of sleep fragmentation and systemic inflammation on brain function. Neuroimaging studies have shown structural and functional changes in the brains of patients with OSA, particularly in areas related to cognitive and emotional processing [3,54].

GLP-1 has been shown to have neuroprotective properties, including the promotion of neuronal survival, reduction in oxidative stress, and enhancement of neurogenesis [18]. Moreover, in animal models, GLP-1 increases non-REM sleep, while sleep deprivation causes GLP-1 and insulin-altered postprandial secretion [75,76].

These effects are particularly relevant in the context of OSA, where chronic intermittent hypoxia can lead to neurocognitive impairments. In addition, rats overexpressing GLP-1R showed improved learning and memory [77], whereas GLP-1 deficiency due to REM sleep deprivation can negatively impact anxiety and depression behaviors [78].

Preclinical studies have demonstrated the neuroprotective effects of GLP-1 in models of neurodegenerative diseases such as Alzheimer’s and Parkinson’s. Clinical trials have also indicated the potential cognitive benefits of GLP-1RA in patients with diabetes [18,29,79]. These findings suggest that GLP-1 may help protect against the neurocognitive deficits associated with OSA.

## 4. Materials and Methods

This narrative review was conducted by searching various databases including PubMed, Scopus, and Web of Science for studies related to GLP-1RAs and OSA. The search was restricted to articles published in English within the last 20 years. The studies included were peer-reviewed articles, randomized controlled trials, observational studies, and relevant meta-analyses. We excluded non-peer-reviewed articles and studies focusing solely on animal models. The primary keywords used for the search were ‘GLP-1 receptor agonists’, ‘Obstructive Sleep Apnea’, ‘OSA management,’ and ‘clinical trials’. The literature was reviewed and selected based on relevance to GLP-1RAs’ role in OSA treatment and its potential benefits on related comorbidities.

## 5. Existing Literature about GLP-1RA and OSA

Several studies have examined the efficacy of GLP-1RA in the treatment of OSA (Table 1).

Liu et al. [83] examined the impact of GLP-1RA on sleep disturbances and diabetic microangiopathy in 93 patients with T2DM and OSA. Participants were divided into a treatment group (50 patients, liraglutide) and a control group (43 patients, conventional drugs). Changes in BMI, waist circumference, HbA1c, blood pressure, lipid profile, uric acid, apnea-hypopnea index (AHI), and microangiopathy were compared over six months. The treatment group showed significant reductions in BMI, waist circumference, HbA1c, systolic blood pressure, and AHI compared to the control group. Additionally, microangiopathy improved significantly in the treatment group [83].

Blackman et al. [81] conducted a 32-week randomized double-blind clinical trial with 276 non-diabetic obese patients, who had moderate (AHI = 15–29.9/h) or severe (AHI ≥ 30/h) OSAS. Participants received either liraglutide (starting dose 0.6 mg/day, increasing weekly by 0.6 mg to a maximum of 3 mg/day) or placebo, alongside diet and exercise advice. The results showed a greater reduction in mean AHI in the liraglutide group (−12.2/h) compared to the placebo group (−6.1/h), with a significant difference (95% CI, −11.0 to −1.2, *p* = 0.015) [81].

O’Donnell et al. [80] conducted a 24-week proof-of-concept study comparing CPAP therapy alone (*n* = 11), liraglutide alone (*n* = 10; starting dose 0.6 mg/day, increasing weekly to a maximum of 3 mg/day), and the combination of liraglutide and CPAP (*n* = 9) in obese participants with moderate-severe OSAS (AHI > 15/h). All treatments significantly reduced AHI compared to baseline (CPAP: 48 ± 20 vs. 3 ± 3; liraglutide: 54 ± 21 vs. 42 ± 16; and combination: 48 ± 17 vs. 5 ± 5; all *p* < 0.05). However, liraglutide alone was less effective (42 ± 16/h) compared to CPAP (3 ± 3/h) and the combination (5 ± 5/h). The authors concluded that CPAP was superior to liraglutide in reducing vascular inflammation [80].

Jiang et al. [82] performed a randomized controlled trial to assess liraglutide in T2DM patients with moderate-severe OSA (AHI ≥ 15/h) on CPAP therapy. Participants received either liraglutide (starting dose 0.6 mg/day, increasing weekly to a maximum of 1.8 mg/day) plus CPAP (*n* = 44) or CPAP alone (*n* = 45) for three months. The combination treatment significantly reduced AHI (26.1 ± 7.1/h) compared to CPAP alone (31.6 ± 6.9/h, *p* < 0.05). The minimum oxygen saturation also improved with the combination therapy (83.4 ± 5.8%) versus CPAP alone (80.4 ± 5.9%, *p* < 0.05). Importantly, patients lost significantly more weight and experienced a significantly greater reduction in systolic blood pressure in the liraglutide group. However, there was no difference in the glycemic control or lipid parameters [82].

Garcia de Lucas et al. [84] explored the therapeutic potential of liraglutide in a 62-year-old male with stage-A1 HIV, T2DM, hyperlipidemia, obesity, and moderate OSAS managed with CPAP. Polysomnography confirmed OSAS with an apnoea-hypopnea index (AHI) of 27/h. After 24 weeks of liraglutide treatment, the AHI was reduced to 7.1/h. The patient also adhered to a regimen including metformin (1700 mg/day), Levemir (30 IU/day), a 1500-calorie diet, and daily walking for an hour [84].

Remarkably, Gomez-Peralta et al. [85] performed a retrospective observational study on 158 obese patients, showing significant reductions in ESS scores at both one-month (baseline: 6.3 ± 4.6 vs. 1 month: 4.9 ± 3.9; *p* < 0.001) and three-month (baseline: 5.7 ± 4.4 vs. 3 months: 4.2 ± 3.6; *p* < 0.001) intervals with liraglutide treatment. The results of a further randomized study with liraglutide are yet to be published [86].

Amin et al. conducted a 4-week randomized controlled trial on the effects of an undisclosed GLP-1RA in participants with moderate OSAS (AHI ≥ 15/h) [87]. Participants were given GLP-1RA (starting dose 0.6 mg/day, increasing weekly to a maximum of 1.8 mg/day) or placebo alongside standard care. The treatment group saw a significant reduction in mean AHI from 50 ± 32 to 38 ± 30 (*p* = 0.0002), with 70% of participants experiencing at least a 44% reduction. Placebo groups did not show significant changes [87].

Idris et al. [88] conducted a 22-week placebo-controlled single-blind study to examine the impact of exenatide on daytime sleepiness in obese patients with T2DM but without an OSAS diagnosis (*n* = 8). Participants received exenatide twice daily (5 µg for the first four weeks and 10 µg thereafter). Daytime sleepiness was assessed using the Epworth Sleepiness Scale (ESS) and the OSLER test. The study found a significant reduction in ESS scores with exenatide treatment (5.7) compared to baseline (12.3) or placebo (11.3) (*p* = 0.003). The OSLER test also showed increased sleep latency with exenatide treatment (37.7 ± 1.7 min) compared to baseline (32.1 ± 1.7 min) or placebo (29.1 ± 1.7 min), indicating reduced objective sleepiness [88].

Wong et al. presented three case studies demonstrating the effectiveness of combining GLP-1RA and pramlintide in treating obesity in patients with type 1 diabetes mellitus (T1DM) [89]. The patients showed significant weight loss and decreased insulin requirements without major side effects.

The results of the SURMOUNT-OSA study have been published recently. The authors conducted two randomized controlled trials. In the first trial, patients without CPAP and in the second trial, patients with CPAP were randomized to tirzepatide (10 or 15 mg) or a placebo. Patients on tirzepatide experienced a greater reduction in AHI than in the placebo group in both trials (−20.0/h, −25.8 to −14.2 95% confidence interval and −23.8/h, −29.6 to −17.9 95% confidence interval; trials 1 and 2, respectively). In addition, tirzepatide significantly improved relevant secondary outcomes, such as body weight, CRP concentration, systolic blood pressure, and sleepiness compared to placebo [17].

Despite the promising results, several studies have reported gastrointestinal side effects such as nausea, diarrhea, constipation, and vomiting, which have occasionally led to non-compliance [80,82]. These studies collectively suggest that GLP-1RA may have a beneficial role in managing daytime sleepiness in patients with metabolic disorders.

A multidisciplinary approach involving sleep medicine specialists, endocrinologists, cardiologists, and dietitians can optimize the management of OSA with GLP-1RA. This team-based strategy ensures comprehensive care, addressing weight management, metabolic health, cardiovascular risk, and sleep quality.

Nonetheless, educating patients about the benefits and potential side effects of GLP-1RA is crucial for enhancing adherence and treatment success. Providing resources such as educational materials, support groups, and regular follow-up appointments can help patients stay motivated and informed about their treatment. Regular monitoring of treatment efficacy and safety is vital. Clinicians should assess weight loss, changes in OSA severity (e.g., AHI), metabolic parameters, and patient-reported outcomes. Adjustments to therapy, such as dose titration or switching to alternative GLP-1RA, should be made based on these assessments.

To conclude, the integration of GLP-1RA into OSA management represents an innovative therapeutic approach, addressing not only the symptoms of OSA but also its underlying metabolic and cardiovascular risk factors. This dual benefit underscores the potential of GLP-1RA to transform the treatment landscape for OSA.

Ongoing and future research should continue to explore the multifaceted effects of GLP-1RA in OSA, focusing on mechanistic insights, long-term outcomes, and personalized treatment approaches. Collaboration across disciplines and leveraging advanced technologies will be key to advancing our understanding and optimizing patient care.

For the successful clinical integration of GLP-1RA in OSA management, a comprehensive patient-centered approach is essential. This includes developing standardized protocols, fostering multidisciplinary collaboration, and providing robust patient education and support. By doing so, we can maximize the therapeutic potential of GLP-1RA and improve the lives of OSAS patients.

## 6. Limitations of the Review

This narrative review has several limitations. First, the absence of adherence to PRISMA guidelines, as it is a narrative review rather than a systematic review. Consequently, the synthesis of evidence may not follow a predefined protocol, which could influence the comprehensiveness of the literature covered. Second, the literature search was limited to articles published in English, which may have led to the exclusion of relevant studies published in other languages. Lastly, the review does not fully capture ongoing or unpublished research, which may provide further insights into the therapeutic potential of GLP-1 receptor agonists in OSA management.

## 7. Future Directions and Conclusions

Even though current research highlights the beneficial effects of GLP-1RA on weight loss and metabolic health, the specific mechanisms through which GLP-1 influences respiratory control and upper airway muscle tone in OSA remain unclear. Future studies should focus on elucidating these mechanisms, potentially through advanced imaging techniques and electrophysiological studies in animal models and humans.

Long-term data on the efficacy and safety of GLP-1RA in patients with OSA are limited. Extended follow-up studies are needed to assess the durability of weight loss, improvements in OSA severity, and cardiovascular benefits. Additionally, the long-term safety profile in patients with OSA, particularly concerning potential side effects and adherence, requires further investigation. Further research should explore the potential synergistic effects of combining GLP-1RA with other OSA treatments, such as CPAP therapy, mandibular advancement devices, or positional therapy. Studies could investigate whether combination therapies offer superior outcomes compared to monotherapy, focusing on adherence, patient satisfaction, and overall treatment efficacy.

Moreover, identifying specific patient subgroups that may benefit the most from GLP-1RA therapy is crucial. Future studies should aim to stratify patients based on factors such as baseline body mass index (BMI), metabolic health, severity of OSA, and presence of comorbid conditions. Personalized treatment approaches could optimize the therapeutic benefits of GLP-1RA in OSA.

The direct effects of GLP-1RA on sleep architecture and quality beyond weight loss need further exploration. Polysomnographic studies could help determine changes in sleep stages, arousal indices, and overall sleep efficiency with GLP-1 therapy, providing a more comprehensive understanding of its impact on sleep health.

Incorporating GLP-1RA into existing clinical guidelines for OSA management requires the development of standardized protocols. Guidelines should outline criteria for patient selection, dosing regimens, monitoring parameters, and follow-up schedules. Collaboration between endocrinologists, sleep specialists, and primary care providers is essential to ensure a cohesive approach.

## Figures and Tables

**Table 1:** Overview of the existing literature examining the efficacy of GLP-1RA in the treatment of OSA.

Study	Participants	Treatment	Duration	Results
Liu et al. [75]	93 patients with T2DM and OSAS	Liraglutide (50 patients) vs. conventional drugs (43 patients)	6 months	Reductions in BMI, waist circumference, HbA1c, systolic BP, AHI, and improved microangiopathy
Blackman et al. [76]	276 non-diabetic obese patients	Liraglutide vs. placebo	32 weeks	Greater reduction in mean AHI in liraglutide group (−12.2/h) vs. placebo (−6.1/h)
Amin et al. [80]	Participants with moderate OSAS	GLP-1RA vs. placebo	4 weeks	Reduction in mean AHI from 50 ± 32 to 38 ± 30 in treatment group
Garcia de Lucas et al. [79]	Case Report	Liraglutide	24 weeks	AHI reduced from 27/h to 7.1/h after liraglutide treatment
O’Donnell et al. [77]	Obese participants with moderate-severe OSAS	CPAP therapy alone vs. liraglutide alone vs. combination	24 weeks	All treatments reduced AHI, with liraglutide alone being less effective
Jiang et al. [78]	T2DM patients with moderate-severe OSA on CPAP	Liraglutide plus CPAP vs. CPAP alone	3 months	Combination treatment reduced AHI and improved minimum oxygen saturation
Sprung et al. [81]	Patients newly diagnosed with OSAS, obesity, and T2DM	Liraglutide alone, liraglutide plus CPAP, CPAP alone	26 weeks	Study proposed
Idris et al. [82]	Obese patients with T2DM without OSAS	Exenatide vs. placebo	22 weeks	Reduction in ESS scores with exenatide
Gomez-Peralta et al. [83]	158 obese patients	Liraglutide	3 months	Reductions in ESS scores at one and three months with liraglutide
Wong et al. [84]	Patients with T1DM	GLP-1RA and pramlintide	7 months	Weight loss and decreased insulin requirements

Abbreviations: GLP-1RA = Glucagon-like Peptide-1 Receptor Agonists; OSAS = Obstructive Sleep Apnea Syndrome; AHI = Apnea-Hypopnea Index; BMI = Body Mass Index; T2DM = Type 2 Diabetes Mellitus; CPAP = Continuous Positive Airway Pressure; T1DM = Type 1 Diabetes Mellitus; ESS = Epworth Sleepiness Scale.
